# Perceptions of Economic Inequality in Colombian Daily Life: More Than Unequal Distribution of Economic Resources

**DOI:** 10.3389/fpsyg.2018.01660

**Published:** 2018-09-06

**Authors:** Efraín García-Sánchez, Guillermo B. Willis, Rosa Rodríguez-Bailón, Juan Diego García-Castro, Jorge Palacio-Sañudo, Jean Polo, Erico Rentería-Pérez

**Affiliations:** ^1^Department of Social Psychology, University of Granada, Granada, Spain; ^2^Sede de Occidente, University of Costa Rica, San José, Costa Rica; ^3^Grupo de Investigaciones en Desarrollo Humano, Department of Psychology, Universidad del Norte, Barranquilla, Colombia; ^4^Instituto de Psicología, Universidad del Valle, Cali, Colombia

**Keywords:** perceptions, economic inequality, framing, content analysis, Colombia

## Abstract

Research on perceptions of economic inequality focuses on estimations of the distribution of financial resources, such as perceived income gaps or wealth distribution. However, we argue that perceiving inequality is not limited to an economic idea but also includes other dimensions related to people’s daily life. We explored this idea by conducting an online survey (*N* = 601) in Colombia, where participants responded to an open-ended question regarding how they perceived economic inequality. We performed a content analysis of 1,624 responses to identify relevant topics and used network analysis tools to explore how such topics were interrelated. We found that perceived economic inequality is mainly represented by identifying social classes (e.g., the elites vs. the poor), intergroup relations based on discrimination and social exclusion, public spaces (e.g., beggars on streets, spatial segregation), and some dynamics about the distribution of economic resources and the quality of work (e.g., income inequality, precarious jobs). We discuss how different perceptions of economic inequality may frame how people understand and respond to inequality.

## Introduction

Despite the significant increase of economic inequality all over the world, people usually overlooked the income gaps between the haves and the have-nots (Norton and Ariely, 2011; Kiatpongsan and Norton, 2014). The lack of accuracy with which people perceive economic disparities is usually associated with lack of information about how economic resources are distributed in society, or even with poor awareness or concern about inequality (Kelley and Evans, 1993; Castillo, 2012). From this perspective, perceived economic inequality is constrained to how individuals estimate income disparities. Indeed, the study of perceived economic inequality has mainly focused on the estimations of income/wealth gaps or on the beliefs associated to how resources are allocated (Janmaat, 2014). However, perceiving economic inequality implies more sophisticated views entrenched in people’s daily life according to their biographical, historical, and contextual repertoires (Irwin, 2016). Considering this, the aim of this study was to analyze how people perceive economic inequality on the basis of their own experience and immediate context. To do so, we intended to identify the topics used by individuals when they perceive economic inequality. Such everyday perceptions have relevant implications on how people understand inequality and respond to it.

We conducted an online survey among university undergraduates to collect open-ended responses regarding how they perceived economic inequality. We combined content analysis and network analysis techniques to identify the most salient topics and the relationships between them. Specifically, we combined a hermeneutic approach to interpret all the elements embedded in participants’ responses with quantitative techniques from the field of network analysis to analyze how the main topics create clusters of meaning that have not been sufficiently covered in previous research about perceptions of economic inequality.

### Perceived Economic Inequality: More Than Income/Wealth Estimates

Perceived economic inequality refers to how individuals see income distribution, focusing on the subjective magnitude of differences in financial resources (Engelhardt and Wagener, 2014). A systematic review about perceptions of inequality has shown that research on this topic has been focused on (a) its distributional representation, which is based on the estimation of income/wealth gaps; (b) beliefs to explain or justify inequality; and (c) judgments about the fairness of such disparities (Janmaat, 2014). In this framework, people are usually asked how much inequality they perceive or accept, what they think about it, or how they judge and evaluate income/wealth disparities. In all these cases, perceived economic inequality is framed as a distributional issue. Thus, people are driven to think about economic resources by being asked to make estimations or judgments about how such resources are allocated.

From this perspective, it has been found that people tend to underestimate the income gaps that exist around them, so that their perceptions do not mirror the true levels of economic inequality (Norton and Ariely, 2011; Kiatpongsan and Norton, 2014; Norton et al., 2014). Although such misperceptions can be due to lack of information or awareness of economic inequality (Kelley and Evans, 1993; Castillo, 2012); people have difficulties being attuned to general or broader societal concepts, so they use their immediate social environments as reference points instead (Galesic et al., 2012). For instance, the wealthy, who move in affluent circles, extrapolate their affluent reality to society as a whole when estimating general levels of wealth (Dawtry et al., 2015). Thus, the study of perceived inequality should also include the analysis of how people use the information available on their social environments.

Additionally, perceived economic inequality is also biased by the ideological climate, social norms, and individual beliefs. Previous research has shown that adherence to liberal political ideologies—compared to conservative ones—can lead individuals to estimate higher levels of inequality (Chambers et al., 2014). What is more, there are other socio-psychological mechanisms through which people overlook and legitimate inequality, such as the acceptance of narratives related to upward social mobility (Shariff et al., 2016), the endorsement of system-justifying ideologies (Jost and Hunyady, 2005), and the promotion of meritocratic discourses (Mijs, 2016). Therefore, perceived economic inequality is not only an estimation issue, but also rather the result of individual repertoires, socio-psychological processes, and contextual issues.

Perceived economic inequality is more than calculating income gaps or wealth distribution; instead, it is a phenomenon that can be perceived and experienced in many different ways that are not exclusive to the economic arena. Rather than abstract and conceptual definitions of economic inequality, people tend to use biographical references to talk about inequality and to posit themselves in such represented social structure (Irwin, 2016). Hence, when studying perceptions of inequality by just asking for how resources are distributed, researchers are leaving behind other indicators about how people understand such inequality, which might have different implications on how people respond to inequality.

### Frames of Perceived Economic Inequality

The concept of framing refers to how descriptions of reality are made from particular perspectives with specific interpretative guidelines (Goffman, 1974). Framing implies the selection and salience of particular aspects of reality in order to promote specific elements to understand, evaluate, and react to it (Entman, 1993). Insofar as framing focuses on certain attributes while ignoring others, it can shape people’s beliefs, motivations, and preferences (Lakoff, 2006). In this regard, seminal research by Tversky and Kahneman (1981), Kahneman and Tversky (1984) demonstrated that the formulation of logically equivalent problems (e.g., presenting problems in terms of gains or losses) highly influences individuals’ decision-making process.

Framing concepts and phenomena can lead to different social judgments about politics (Druckman and Nelson, 2003; Lee et al., 2008; Sides, 2016). For instance, studies have shown that framing groups of people as victims (Moscovici and Pérez, 2007), as underdogs (Vandello et al., 2007), or as people in need (Shnabel et al., 2016) led dominant groups to be more willing to support them in several ways. In such cases, framing triggered guilt, empathy, or compassion among individuals, eliciting pro-social behavior among them even at expense of their own self-interest (Lowery and Wout, 2010).

Although economic inequality involves both the haves and the have nots, it can be framed by emphasizing either the advantaged or the disadvantaged side (Wänke and Reutner, 2011). Focusing on one side or another has different effects on how people understand and react to inequality. For instance, framing economic inequality as people having more than the average (e.g., the rich have more than…) led conservatives to support heavier taxation for the rich (Chow and Galak, 2012), average individuals to delegitimize economic inequality (Bruckmüller et al., 2017) and support measures that take more resources from the rich (Lowery et al., 2009).

Kahneman and Tversky (1984) posited that the frames used to perceive and assess some issues can be seen as different perspectives with which the same reality can be approached. Based on this, the distributional way to frame inequality—“having more than” or “having less than”—is just one of the perspectives but cannot delimit the whole phenomenon. Experiments on framing inequality are useful to analyze this specific perspective; but such frames mostly represent the distributional idea, and do not include the complex network of ideas and experiences that people might have in mind when they think about economic inequality in their real life. Indeed, people are not constantly exposed to frames about “having more/less than.” Instead, individuals navigate through their daily life while trying to make sense of their realities according to their experiences and the context that surrounds them. Therefore, focusing on how people perceive inequality on a daily basis is likely to provide other ways to frame it, contributing to a better understanding of what people are paying greater attention to and how they react to this issue. Nevertheless, to our knowledge, these alternative frames of economic inequality have not been covered enough in the empirical literature. Our research was intended to bridge this gap by exploring the different frames associated with perceived economic inequality and setting a starting point for future research to explore its potential implications.

In this vein, the aim of this study was to identify how people perceive economic inequality in their daily life. We propose that perceived economic inequality covers a broad variety of dimensions of individuals’ daily life rather than only the estimation, belief, or judgment about how economic resources are distributed. This was intended to shed light on the various frames usually used by people when perceiving economic inequality. Thus, this research contributes to the literature in the field by showing that economic inequality is perceived beyond the economic resources dimension; by illustrating how network analysis techniques can boost qualitative data analysis; and by providing a public data corpus about perceptions of inequality in Colombia that can be used to answer further research questions.

## Materials and Methods

### Participants and Data Corpus

We invited undergraduates in 12 Colombian universities to participate in an online opinion survey about social and economic issues. The sample was composed of 601 undergraduates^1^ (*M*_age_ = 21.71, SD = 4.11; 65.56% female, 29.62% male, 4.82% unreported), who successfully answered the following open-ended question: “*How do you perceive economic inequality in Colombia? Please write down up to 3 responses.”* Although participants were undergraduates, they reported being from diverse social and economic backgrounds: a total of 62.91% were enrolled at a private university, and 37.09% at a public university; universities were located in five cities (Barranquilla, Santa Marta, Cartagena, Cali, and Palmira) from two different regions of Colombia (South-west and Caribbean), yet participants reported to live in 19 cities along those regions, under different socioeconomic strata^2^ (14.69% in strata 1, 21.70% in strata 2, 37.40% in strata 3, 13.02% in strata 4, 8.35% in strata 5, and 3.67% in strata 6), and along different household incomes (7.81% up to the one minimum wage—MW—, 25.35% between 1 and 2 MW, 30.03% between 3 and 5 MW, 19.10% between 5 and 7 MW, 9.38% between 7 and 9 MW, 4.86% between 10 and 12 MW, and 3.47% more than 12 MW). There were three open calls to participate in the study, one every week. Data collection took place between 20 September and 26 November 2016.

We obtained an average response rate of 2.7 answers per participant, and our final set of data was composed of 1,624 responses, resulting in a data corpus of 21,968 words^3^. Participants were enrolled in various schools, including those of psychology, management, engineering, and health sciences. Data were processed by using content analysis techniques based on grounded theory (Strauss and Corbin, 1998). Given that we had no previous categorical framework available beforehand, we built conceptual categories directly from participants’ responses. Our categorical framework was composed of different *topics* that were related to various subjects or contents (e.g., social actors, basic services, work). We also included a category to mention the *sense* in which the topics were being used. Specifically, people can mention the same topic (e.g., health) but in different senses (e.g., lack of access = just the disadvantaged; or unequal access = both the advantaged and disadvantaged) (see **Table 1** for the categorical framework).

**Table 1:** Categorical framework^∗^.

Category (with definition)	Subcategory
**Basic services:**	
Primary services to survive and have a dignified life	• Access to basic services (general) • Food • Health • Public transport • Housing
**Living conditions:**	
Physical characteristics, conditions, or any material/social elements that define a way of living	• Economic resources concentration • Living conditions (general) • Criminality or insecurity • Forced displacement • Public space • Social stratification • Pensions • Income • Predatory loans (banking or not) • Rural sector • Social subsidies
**Poverty:**	
State of having little, too few or any money, goods, or means to live; also considered as not having enough of anything that is considered as necessary	• Begging • Homeless people • Poverty (general)
**Affluence:**	
Having abundance of money and material goods	• Affluence or opulence (general) • use of expensive goods and services
**Opportunities:**	
Conditions to do or achieve something, it includes opinions regarding getting ahead in life	• Education • Meritocratic beliefs • Inequality of opportunities (general) • Opportunities in life (general)
**Consumption:**	
Use, buy, or just have access to certain products or services	• Saving • Consume products or services • Queues to have access to services • Leisure
**Work:**	
Any economic productive activity through which people make a living. It includes both employments as other forms of work (cooperative, informal, independent, etc.)	• Economic migration • Child labor • Informal work • Career • Unemployment • Access to work • Work (general) • Precarious work
**Institutional issues:**	
Related to social, economic, or political institutions. Not as actors (e.g., politicians), but as the system that represents	• Taxes • Public investment • Justice • Media • Political system
**Interpersonal relationships:**	
Focused on how people relate to each other	• Treatment of people • Social comparisons • Social conflicts • Ethnic or cultural issues • Family
**Social actors:**	
People, groups, organizations, institutions, or any social category that represent a figure with a specific role in the social dynamic	• Older people • Banks • Peasants • Social classes (general) • Directives, Chiefs, Bosses, supervisors • Elites • Enterprises • Private entities • Public entities • Students • Public servants • Government • Youth people • Women • Children • Poor • Police • Society (general) • Workers • University • Private university
**Senses:**	
Way to use the topics identified. The senses try to identify the intention attributed to each category (when possible)	• Economic activity • Corruption • Unequal access • Inequality of physical conditions according to zones • Inequality in living expenses • Gender inequality • Income inequalities • Misinformation • Lack or difficulties to access • Mobility difficulty • Lack of economic resources • Privatization • Spatial segregation • Discrimination

*^∗^Detailed indicators per subcategory can be found in **Supplementary Table S1**.*

### Procedure

We used content analysis due to its usefulness to identify and systematize structures embedded in the language (Bardin, 2002). Our recording unit—the minimum part of our body of data with meaning—was each single *response*. The enumeration rules used for processing the data were *frequency*—amount of times that each category appeared in the data corpus—and *co-occurrence*, that is, the presence of two or more categories in the same recording unit. The coding process was supported by Atlas.ti 7.5.18 software and network analysis techniques were conducted with Gephi 0.9.2 software. Network analysis provides a set of techniques to understand relationships among, between, and within people, groups, or as in our case, concepts (Hanneman and Riddle, 2005). We examined the configuration of the overall relationships between all the topics and identified several clusters of meaning. In this sense, we combine a hermeneutic approach by interpreting each response; and a quantitative approach to identify prevalence topics and to disentangle how all topics were associated among them.

Five coders classified the responses through a four-stage process. First, we carried out three training sessions to code the responses of 30 participants by coding part of the information together and leaving individual coding work to be discussed during the training sessions. Second, once we had obtained a set of preliminary categories, the main researcher coded all the data and ended with a more specific categorical framework (see **Table 1**). Third, all the coded data were divided among coders to review the coding. Here, the focus was on finding ambiguities, missing, or excessive coding. The fourth step was to resolve the issues raised in the reviewing process by reaching agreements between researchers on each coded response. Because of the purpose of this research was exploratory, we did not have a pre-established categorical framework to do the coding, but we construed it during the research. Therefore, the reliability of the coding is based on the deliberative agreement among researchers, instead of statistical measures of inter-rater reliability. This means that every piece of coded information was reviewed several times by at least two researchers, which allowed us to control for potential biases coming from a single coder.

The coding was done taking into account the following statements: (1) all the categories present in each response had to be identified; (2) coding should be based on literal text, avoiding an over-interpretation of responses; (3) the reviewer should explicitly find the reason why a response was coded in a specific category, otherwise such coding would be dismissed; and (4) potential ambiguities in the coding would be solved by reaching intersubjective agreement on the basis of explicit responses. **Table 2** illustrates how each response was treated.

**Table 2:** Example of the coding exercise.

Quotation 31:3		Coding
*In the mall and other commercial establishments*	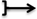	Conditions: public space Interpersonal: treatment of people
*there is a higher predisposition against*	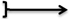	Senses: discrimination
*the people of low status*	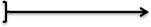	Actors: social classes, poor

## Results

This section is divided into two parts. In the first part, we present a frequency analysis of the main contents observed in our data corpus, which enabled us to identify common and specific topics used when people perceive economic inequality. In the second part, we do a co-occurrence analysis by using network analysis tools to depict the relationship between categories.

### Perceptions of Economic Inequality: A Matter of Social Classes, Public Spaces, and Work

As shown in **Table 3**, the most frequent topics were *education*, *social classes* (including both “the elites” and “the poor”), *income*, *health*, and *unequal* and *lack of access to some goods and services*. These topi These topics confirm the results of previous studies (Smart, 2012; Flanagan et al., 2014) that have shown the central role of social categories based on socioeconomic status, such as “rich” and “poor.” However, such distinction is not based exclusively on the concentration of wealth (*income*), but also on the possibility of having access to better educational and health services. Individuals particularly denoted that such disparities are due to an unfair system that favors one group (e.g., *elites*) while excluding and discriminating the other (e.g., *poor*).

**Table 3:** Frequencies and percentages of responses coded in each category.

Category	Frequency^∗^	Percentage^∗∗^
Opportunities: education	304	18.72
Actors: social classes (include elites and poor)	274	16.87
Living conditions: income	267	16.44
Basic services: health	257	15.83
Senses: unequal access	216	13.30
Senses: lack or difficulties to access	192	11.82
Actors: workers	179	11.02
Living conditions: public space	163	10.04
Senses: discrimination	150	9.24
Actors: elites	139	8.56
Senses: income inequalities	121	7.45
Work: precarious work	119	7.33
Actors: poor	96	5.91
Interpersonal: social comparisons	95	5.85
Interpersonal: treatment of people	94	5.79
Living conditions: socioeconomic stratification	89	5.48
Work: work (general)	85	5.23
Senses: corruption	83	5.11
Actors: university	82	5.05

*We set an arbitrary cut-off point at appearance frequency of 5%, but full information can be found in **Supplementary Table S2**. ^∗^Number of responses coded in each category. ^∗∗^Percentage is computed on the basis of total responses of our data corpus (N = 1624).*

Low-strata people do not have access to high quality education, while people who have money can afford the best schools (82:2^4^)

Only the rich can have access to high quality health and education (642:2)

Another topic frequently used was *public spaces*, which indicates the place where people see and experience economic inequality. The main public spaces mentioned by respondents were the streets, avenues and the city center, where they usually see beggars, informal workers, and homeless people. Neighborhoods were also relevant places where individuals recognized economic inequality but, in this case, they pointed out the *spatial segregation*, noting that some neighborhoods have optimal living conditions (e.g., parks, roads, cleaning services), whereas others do not. In addition, malls, parks, shops, and other commercial hubs were depicted as places where people can consume goods or services according to their wealth, that is, some have access to leisure activities and fancy items, while others do not have access to food, clothing, or shelter.

The difference between neighbourhoods (…); high-strata neighbourhoods are idyllic places, everything is beautiful, nice streets, mansions, lakes, trees, restaurants, shops, malls; but in the same city there are also low-strata places that are full of burglars, *sicarios [hitman]*, physically and mentally sick people, prostitutes, drug dealers, it’s such a terrible area (…) (745: 3).

Other relevant topics were related to the world of work: *workers*, *income inequalities*, *discrimination*, and *precarious work*. These topics highlight the role of work as an activity associated with social inclusion/exclusion. In fact, work is a productive economic activity by means of which people can obtain decent living conditions not only through their earnings but also through decent working conditions. *Precarious work* refers to poor working conditions (i.e., unfair salaries, high income gaps, long working hours, and various types of labor exploitation) that prevent individuals from having a decent life through having access to social protection and prospects of personal development.

The salary of a domestic worker [female] is too low compared to that of a professional and, honestly, the former works much harder than the latter (57:1).

Congressmen earn more than 25 million pesos just for sitting in Congress and people working in the streets under the sun and harsher conditions hardly earn the minimum wage (82:1).

### Clusters of Meaning About Perceived Economic Inequality

We used network analysis tools to explore the relationship between the different categories coded in our data corpus. Given that simple co-occurrence analyses only allowed us to see one-to-one relationships, it makes difficult to handle large amount of co-occurrences. Thus, we decided to take advantage of network analysis tools to depict all the relationships at the same time. Thus, using the co-occurrence matrix, we created a list with each combination of two categories and the number of responses that it contained. This information enabled us to analyze and visualize the relationships by using a set of graphs, including the quantitative metrics commonly used by network analysis techniques.

Graphs are created on the basis of matrix theory principles (Diestel, 2010), and are made up of two main components: nodes, which represent each category, and edges, which show the type of relationship between nodes. In our case, the edges were the amount of responses that contained each pair of categories, so that higher values meant higher co-occurrence. Whereas frequency analysis gave us an idea of the most common topics mentioned in our corpus, network analysis provided a different perspective by depicting how those categories were interconnected.

To analyze such networks, we focused on two centrality metrics. One of them was the *degree of centrality*, that is, the amount of relationships that each node has with other nodes. Thus, a higher degree of centrality indicates more relationships with other nodes, and therefore, more relevance in the network (Brandes and Patrick Kenis, 2005). Another metric we used was betweenness centrality, which represents the extent to which a node lies along the shortest path connecting others in the network (Park and Leydesdorff, 2013). In other terms, the degree of centrality tell us what are the more connected nodes; and betweenness centrality helps to identify the most influential nodes in the network.

**FIGURE 1:**
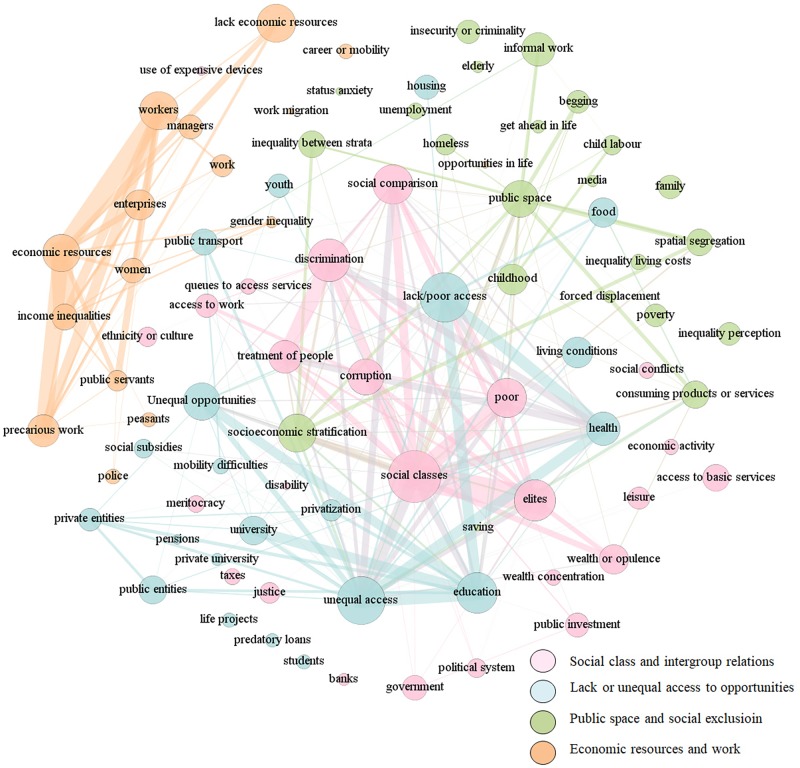
Perceptions of economic inequality graph based on co-occurrence matrix with clusters marked in colors.

The final network included 84 nodes, matched in 908 one-to-one relationships, which ranged from 1 to 139 times of appearance. We obtained a thick network (*density*^5^ = 0.26), which indicates that the categories were highly interconnected. To visualize the data, we used the layout proposed by Fruchterman and Reingold, which is a spring-embedded algorithm based on the gravitation of nodes according to the attraction or repulsion to other nodes. The result is a force-directed network where the most relevant nodes are bigger in size (higher degree of centrality); the most strategic nodes are placed in more central positions; the most related nodes are closer to each other (betweenness centrality); and the thickness of the links reflects the strength of each relationship (amount of times that such relationship appeared in our corpus) (Cherven, 2015). In order to identify clusters of meaning in this graph, we applied a modularity class algorithm available in Gephi software (Emmons et al., 2016), which is based on a modularity statistic that identifies different groups of nodes according to the strength of their relationships (Cherven, 2015). In our graph, we visualized the identified clusters by colors. Due to the high density of the graph, we plotted all the nodes, but only showing relationships above 14 co-occurrence times^6^ to facilitate the interpretation. **Figure 1** presents the whole graph; full network metrics are available in **Supplementary Table S3**.

### Hotspots of Perceived Economic Inequality: Social Classes, Discrimination, and Lack or Unequal Access to a Decent Life

In terms of the betweenness centrality metric, we found that categories with the greatest likelihood of connecting with other topics were the *lack of access or poor access to goods or services*—mainly health and education—, *social classes*, and *discrimination toward the poor*. In other words, these categories function as bridges through which other topics relate to each other. Thus, the core topics we identified are not based on the idea of how economic resources are allocated, but rather on how groups of people do not have access to decent living conditions, and how corruption leads privileged groups to discriminate the disadvantaged because of their social background.

The *public space* is another linking category that brings together concepts such as *socioeconomic stratification*, *spatial segregation*, and the prevalence of *informal work(ers)* on the streets. Again, economic inequality was placed in the public arena of daily life, where social comparisons can be particularly salient, stressing intergroup tensions. Though present, mentions of *wealth concentration*, extreme affluence, or any other abstract meaning of inequality were barely mentioned when compared with other codes.

In addition, the modularity class algorithm we used in network analysis let us identified four clusters of categories that we labeled according to the centrality of its nodes: social class and intergroup relations, public space and social exclusion, lack or unequal access to opportunities, and economic resources and work. These clusters are not mutually exclusive, but rather the opposite they were highly intertwined; such that the more central nodes in each cluster were highly associated between them. However, the cluster related to economic resources and work (in orange color) set apart from the others. This distinction seems to represent two different domains regarding how economic inequality can be perceived: based on how economic resources are distributed, or as daily life experiences.

#### Social Class and Intergroup Relations

The first cluster grouped 29.76% of the nodes and was centered on the *social class* category, which refers to the *elites* and the *poor*. These social actors are mainly associated with intergroup categories, such as the *treatment of people*, *social comparisons*, and *discrimination*. Economic inequality seems to trigger the idea of intergroup relations that underline the differences and discrimination between groups based on status indicators. Particularly, such social comparisons and discrimination are made in terms of having—or not having—access to basic services, such as health or education. Discrimination of the disadvantaged and favoring of the advantaged is linked to corrupt practices that are overtly institutionalized by the system. In fact, individuals recognize a system that maintains and reproduces inequalities by providing social services according to people’s socioeconomic background.

In a hospital, if low-strata and high-strata patients arrive at the same time, most of the time they take care of high-strata ones first (139:1).

In the social strata, people with money discriminate the poor just because they do not have money, without knowing anything about their situation (330:3).

#### Public Space and Social Exclusion

The second cluster grouped 25% of the nodes and focused on public space, which was seen as the main scenario where participants could witness economic inequality. This cluster was mainly related to the places and situations where economic inequality takes place, such as spatial segregation, socioeconomic stratification, informal work, and overt poverty, begging, and homeless people living on the streets.

It is worth noting that Colombia has an official division of the public space into socioeconomic strata. Urban spaces are classified according to the accessibility to certain public services, the quality of overall living conditions and therefore the purchasing power of the people who live there (socioeconomic strata range from 0 to 6). This public policy was designed to charge public services according to the purchasing power of citizens. However, that distinction has become a social category, so people use it as an identity reference (e.g., “I’m strata…”). In fact, people use a spatial label to categorize both themselves and the others. The mention of socioeconomic strata also is related to a spatial segregation system, given that some people have access to better living conditions (e.g., roads, schools, security) just because they are part of an elite; by contrast, people at the bottom hardly have decent living conditions. These perceptions increase the discrimination shown in intergroup relations and recognize broader institutionalized practices of exclusion.

Inequality of strata; the best is for super rich people; even though we have the best attitude, the doors are closed to us (345:3).

In the neighborhoods of Cali, because people are categorized as rich or poor according to the area where they are living in (527:3).

*Informal work* is associated with people performing different types of economic activities in the public space (e.g., street vendors) without any type of benefits (e.g., health, pension). This topic is intrinsically related to poverty representations, where individuals highlighted the lack of economic resources to have a decent job and life. In this cluster, respondents focused on the disadvantaged side of inequality, downplaying the social comparisons with advantaged groups. Economic inequality was perceived as not having enough, instead of on the unequal distribution of money, goods, and services; this is a perspective that can easily downplay the role of other groups and institutions that contribute to inequality dynamics.

When I go to the university and find adults, adolescents and kids in the streets, cleaning windshields or selling candies instead of working in companies with fair salaries or getting an education (514:1).

The amount of poverty that can be seen in the streets, people begging, kids working in the streets, homeless people (634:3).

#### Lack or Unequal Access to Opportunities: Education, Health and Living Conditions

The third cluster grouped 25% of all the graph nodes and was based on *unequal* and *lack of access* to goods or services. We distinguish between these two categories because each of them represents a different way of framing inequality. Unequal access implies a relational issue, whereby some people can get what they need/want, while others cannot; by contrast, *poor access* focuses on one side of the relationship, namely on those who cannot have it. These two senses were mainly associated with *education*, *health*, *food*, *housing*, and *mobility*. All these elements represent the lack of opportunities in getting the resources needed to get ahead in life. When perceiving economic inequalities, participants were quite sensitive to the relevance of the cultural capital in sharpening intergroup differences. We found that people were aware of the link between different socioeconomic backgrounds and access to social resources (e.g., education, health, mobility) to achieve their goals.

EDUCATION: In Colombia not all of us have the opportunity to reach the same education level; when we finish high school the vast majority of young people are left behind because of not having any opportunities… In Colombia you don’t study what you want, but what little the State can offer you (60:1).

#### Economic Resources and Work: Income Inequality and Precarious Work

This cluster grouped 19.05% of all nodes, was the smallest one found in the whole graph (19.4% of all nodes) and obtained the lowest centrality measurements when compared to the other clusters. This cluster was mainly related to lack of or unequal access to economic resources, and work precariousness. Thus, respondents particularly highlighted how workers perform their work under precarious conditions, such as low wages, large income gaps, long working hours, among other ways of labor exploitation. The category workers was highly mentioned (179 times) in the whole graph, and used to make an argument about *workers* in badly-paid jobs in a context of large income inequalities. *Companies* and *managers* were depicted as relevant factors that determine such precarious work conditions. In addition, people also perceived large gender inequalities, with women being a highly vulnerable social group. Although participants recognized income gaps as an important issue when thinking about economic inequality, this idea was more linked to precariousness at work than to the systemic unequal distribution of economic resources in general.

Breadwinning mothers have several part-time jobs, and even adding up all their earnings they are not enough to have a decent life or to satisfy their basic needs and those of their families (102:2).

Income inequality between men and women in many organizations; women are usually paid much less (273:3).

The seemingly lower appearance of the distributional frame of economic inequality is consistent with previous studies that have found that people use closer and daily life references to understand abstract phenomena (Imhoff and Brussino, 2015). In fact, people bear in mind more vivid images of such inequalities at the expense of omitting other aspects related to systemic economic inequality, such as wealth concentration and income disparities.

## Discussion

The aim of this paper was to examine the perceived economic inequality in the daily life of a sample of undergraduates in Colombia. Based on a data corpus extracted from open-ended responses, we analyzed the topics individuals mentioned when perceiving economic inequality. We found that rather than explicit perceptions associated with income gaps and unequal wealth distribution, participants were more focused on a wide variety of ideas related to intergroup, institutional, and spatial dynamics. Thus, perceptions of economic inequality are not just related to the conceptual definition associated to the distribution of economic resources, but are a complex phenomenon entrenched in people’s daily life (Irwin, 2016).

We found that perceptions of inequality were mainly associated with intergroup and interpersonal dynamics. Participants focused on several social actors (e.g., social classes, elites, the poor, workers) based on socioeconomic status indicators. They also provided recurring examples of discrimination toward the poor, social exclusion and deprivation of decent living standards. Participants also recognized how inequality is reproduced by social institutions such as the health and education services, by favoring high-status people at the expense of others.

The clusters identified in the network analysis complement the idea of economic inequality as something broader than a mere distributional issue. Social classes and intergroup relations, public space, social exclusion, unequal opportunities, and work(ers) and income inequality, as a whole, are a richer discursive repertoire through which people perceive and make sense of such a complex phenomenon. Since most research on perceived economic inequality concentrates on how resources are allocated, we contribute to recognizing the other ways in which people spontaneously perceive and frame economic inequality based on their own experiences.

Our findings contribute to the research on perceptions of inequality in at least three ways: first, perceived inequality does not rely exclusively on a rational thinking process (e.g., gap estimations), but also on social comparison processes (e.g., relationship with their immediate contexts). Second, we provide empirical evidence of other dimensions of perceived inequality that have been overlooked in the literature. And third, though we did not pretend to generalize our findings to other contexts, we consider that the topics we identified might not be constrained to the Colombian context. Indeed, participants’ perceptions mirrored the consequences of widespread neoliberal policies implemented worldwide (e.g., social spending reduction, privatization of health and education services, work precariousness). However, more research should deepen about how perceptions of inequality vary along different contexts, and what might be the implications of such perceptions on people responses to inequality.

Considering the relevance of the context for perceived economic inequality, it is worth noting that the current research was conducted in Colombia, which is one of the most unequal countries in the world. This provides a special case of study due to its long-lasting unequal distribution of income, wealth, land ownership, gender inequality, racial discrimination, and spatial segregation (Salazar, 2009; Valencia and Cuartas, 2009; Acosta, 2013). Moreover, the Colombian context is characterized by a progressive system of privatization of public services as a result of neoliberal policies (Rivillas García et al., 2014; Rodríguez, 2016). It has an official socioeconomic stratification of society (Rosero, 2004; Alzate, 2006) and high rates of people at risk of social exclusion (Valencia and Cuartas, 2009; Vargas et al., 2016). Given such a context, social inequalities tend to be represented as factual and isolated images, without a clear integration with more abstract societal issues (Amar et al., 2001; Amar et al., 2006); our findings corroborate this idea, so that perceived economic inequality is based on daily life experiences rather than an on an abstract/technical definition of unequal distribution of resources.

Additionally to contextual factors, participants’ background is also relevant to how economic inequality is perceived. For instance, is likely that undergraduates have a political socialization that makes them more aware of the inequality of opportunities; whereas the general population might be more sensitive to economic and work issues. Although we cannot generalize our findings to the general population, we consider that our sample is not restricted to a privileged group, but it was diverse enough to represent a wide variety of groups of the Colombian society. Indeed, participants reported coming from different socioeconomic, cultural and geographical backgrounds. This has been possible due to policies offering forgivable loans to disadvantaged communities for accessing to higher education (e.g., “Ser pilo paga” program [being-smart-pays-off]). Further research should explore if the dimensions of perceived inequality we showed in this research are more salient for some individuals according to their social or ideological backgrounds.

Perceived economic inequality in distributional terms is not usually linked to what should be done (mainly in terms of support for public policies) to handle it. As shown in previous research, the mere perception of income inequality does not translate into higher support for policies aimed to reduce it (i.e., progressive taxation, social insurance, regulating economic markets) (Bartels, 2005; Kuziemko et al., 2015). Such seeming disconnection between perceived income gaps and support for redistribution is not just due to people misperceptions—lack of accuracy or concern of inequality—but because of either a poor understanding of how inequality works (Bartels, 2005); or by how such inequality is perceived. Research has demonstrated the focusing on the haves led people to support measurements that take away resources from the rich; whereas focusing in the have-nots was related to providing the poor (Lowery et al., 2009; Chow and Galak, 2012). Thus, responses to inequality are not only a matter of how much inequality is perceived (size) but how it is perceived or framed in peoples’ mind.

Perceived economic inequality can have different implications depending on how people understand it in everyday life. Our findings raise some questions about the potential implications of daily-life frames of economic inequality. First, the proximity to inequality in daily life can trigger negative emotions (e.g., moral outrage, anger, unhappiness) that can lead individuals to engage in collective actions (Tausch et al., 2011). The fact of perceiving inequality in the closest social circles might help individuals recognize all the diverse ways in which inequality is reflected in daily life and, as a result, raise awareness about the problem and motivates people to mobilize against inequality. However, if such vivid perceptions are framed in terms of not having enough, addressing inequality would be a matter of providing more to people in need rather than redistributing resources in the whole society. By contrast, if economic inequality is framed in terms of distribution of economic resources, it might create a greater psychological distance from the phenomenon, becoming something either too abstract or uncommon for people’s reality; which is likely to lead to disengagement from these topics (Liberman and Trope, 2014). In this regard, framing social phenomena in such a way that elicits the unfairness linked to other structural factors is likely to motivate people to engage in collective action (Sabucedo et al., 2017).

Therefore, instead of favoring some inequality frames over others, we argue that it would be necessary to pursue a better integration between the way individuals represent the distribution of economic resources, and their daily-life perceptions of inequality. This might help people to connect their immediate situation with broader social, political, and economic factors. If people can link their daily-life perceptions of inequality to more structural dimensions, they are likely to be able to make better political decisions to tackle this problem (e.g., support for redistributive policies, progressive taxation). However, the analysis of the implications of such different ways of perceiving economic inequality on political decision is out of the scope of this paper and should be addressed in future research. It would be important to provide better evidence about how to improve the impact of social campaigning and engage policymakers and citizens to tackle this topic both in their daily life and in the broader political arena.

## Data Availability

Both the raw dataset generated for this study, and processed data for frequency and network analysis are publicly available in the Open Science Framework platform https://osf.io/m39yv/?view_only=b4194237bb334a4a9368331b1cde048f.

## Ethics Statement

This study was carried out in accordance with the guidelines stated by the Vicerectory of Research and Scientific Policy of the University of Granada, fulfilling the requirements of informed consent and data protection stated by the Spanish Organic Law 15/1999. The protocol was approved by the Ethics Committee for Research of the University of Granada (No. 170/CEIH/2016). This study was conducted using an online platform. Hence, all participants were informed in writing about the objectives of the study, and signed their consent to voluntarily participate in the study. Once the study was concluded, we provided feedback to all respondents regarding the research findings.

## Author Contributions

This research was conducted as a team. Particularly, EG-S, GW, and RR-B contributed conception and design of the study. JP-S, JP, and ER-P contributed with data collection. EG-S and JG-C processed data. EG-S coordinated data collection, performed analysis, and wrote the first draft of the manuscript. All authors made substantial contributions to manuscript revision, read and approved the submitted version.

## Conflict of Interest Statement

The authors declare that the research was conducted in the absence of any commercial or financial relationships that could be construed as a potential conflict of interest.

## Footnotes

1 Out of 794 participants who replied to the call.

2 Colombia has an official socioeconomic stratification system that categorized public spaces ranging from 1 “low-low” to 6 “high-high” according to households purchasing power and value of property. For more information see http://www.dane.gov.co/index.php/69-espanol/geoestadistica/estratificacion/468-estratificacion-socioeconomica.

3 The whole data corpus is available at: https://osf.io/m39yv/?view_only=8823c6515ce6404a8c38257a36a58760.

4 Numbers identify the participant and his/her response number.

5 Graph density is a measure of how interconnected a network is, calculated as the ratio between the edges displayed in the network and the total of all possible connections (Cherven, 2015).

6 This is an arbitrary cut-off point based on 10% of the maximum co-occurrence limit.
