# Supplementary material for: Perceptions of Economic Inequality in Colombian Daily Life: More Than Unequal Distribution of Economic Resources
Source: Front Psychol. 2018 Sep 6;9:1660. doi: 10.3389/fpsyg.2018.01660 (PMC6135891; doi:10.3389/fpsyg.2018.01660)
Supplement: Supplementary file 1 [file Table_1.DOCX]

Supplementary Material

**Perceptions of economic inequality in Colombian daily life: More than unequal distribution of economic resources**

**Efraín García-Sánchez, Guillermo B. Willis, Rosa Rodríguez-Bailón, Juan García-Castro, Jorge Palacio-Sañudo, Jean Polo, Erico Rentería-Pérez**

*** Correspondence:** Corresponding Author: [egarcias@correo.ugr.es](mailto:egarcias@correo.ugr.es)

| **Table S1.**  **Categorical framework with subcategories and indicators** | | |
| --- | --- | --- |
| Category | Subcategories | Indicators |
| **Social actors:** Mention to groups, organisations, or institutions that represent any figure with a specific role in the social dynamics | Older people | Mentions to old people, senior, old-age, ancient |
|  | Banks | banks, commercial banks, credit union, lending institutions |
|  | Peasants | Farmers, people that work in agriculture |
|  | Social classes (general) | Mention to social classes, social categories based on status, affluence, poverty, work, etc. |
|  | Directives, Chiefs, Bosses, supervisors | People that perform roles of power inside an organisation of work |
|  | Elites | High social class, privileged groups, people or groups with more economic, social, and cultural resources. Also people with role of power, such as politicians |
|  | Enterprises | organisations of work, public or private, that plays a role in the society |
|  | Private entities | Mention to private organisations |
|  | Public entities | Mention to public organisations |
|  | Students | People that are in formal education process |
|  | Public servants | People that work for the State or that performs in the official public sector. This is different from the politics mention in the elites categories. |
|  | Government | Mention to government entities or to the government as a whole. |
|  | Youth people | Young people, adolescent, young adults, etc. |
|  | Women | Women in general or in particular |
|  | Children | Children, kids, infants, etc. |
|  | Poor | Low social class, disadvantage groups, defined by the lack of optimal living conditions |
|  | Police | Mention to police, both at the societal level, as well as the public servants |
|  | Society (general) | Mention to society in general terms |
|  | Workers | People that live from their work, no matter if they are into a formal employment or with different work modalities. |
|  | University | Higher education system, universities |
|  | Private university | Private universities or private higher education organisations |

*(Continued)*

**Table S1.** *(Continued)*

| Category | Subcategories | Indicators |
| --- | --- | --- |
| **Living conditions:** Mention to characteristics, conditions, or any material or social characteristic that define a way of living | Economic resources concentration | Mention to wealth concentration, action to hoard many economic resources |
|  | Living conditions (general) | General mention to living conditions without specifying anything |
|  | Criminality or insecurity | References to crime, victimization rates,, insecurity, or any other illegal action |
|  | Forced displacement | Fact of people that need to flee their homes because of security or conflict reasons |
|  | Public space | Any reference to public spaces, such as streets, parks, avenues, outside supermarkets, malls, entertainment and cultural centres, or any other space that can be used by the people. |
|  | Social stratification | Direct reference to the Colombian socioeconomic stratification system. |
|  | Pensions | Pensions, old age benefits, subsidy for older people, retirement income, etc. |
|  | Income | Income, earnings, wages, salaries, or any other economic resource obtained due to the work. |
|  | Predatory loans (banking or not) | loans given by the financial system that are considered as abusive, excessive, unfair, exorbitant, etc. |
|  | Rural sector | Mentions to rural areas, the fields, the country side, etc. |
|  | Social subsidies | any government subsidies to fund or help disadvantage groups |
| **Consuming:** To have access to certain products or services | Saving | The action of saving money |
|  | Consume products or services | Consume any products or services, the fact of consume in itself |
|  | Queues to have access to services | Related to the way how people get access to certain products or services, the queue for getting access |
|  | Leisure | consume of leisure activities, entertainment, free time, etc. |
| **Institutions**: Related to social, economic, or political institutions. Not as actors, but as the system that represents | Taxes | Taxation system, fact of paying taxes |
|  | Public investment | How the government or the political system is distributing the public budget among the people, regions, activities, etc. |
|  | Justice | Justice system, how justice is managed in some places or cases; we also considered the responses where justice was used as a moral code or as personal judgments about fairness. |
|  | Media | Any media, their role on the information of the different situations of the country |
|  | Political system | In general, the whole political system, applies also when not specific institution is mention but make a response about the role of the system. |

*(Continued)*

**Table S1.** *(Continued)*

| Category | Subcategories | Indicators |
| --- | --- | --- |
| **Interpersonal relationships:** Focus on how people relate to each other | Treatment of people | Any kind of interpersonal relations that might well be related to customer service or daily life interactions |
|  | Social comparisons | Explicit comparisons between groups; use of social categories assigned to certain groups to evaluate and compare groups. |
|  | Social conflictiveness | Social problems, coexistence inconvenient, violation of human rights, or any other action that reflects conflicts among people or groups. |
|  | Ethnic or cultural issues | Ethnic, cultural, minority groups, such as indigenous, afro, gypsies, etc. |
|  | Family | Familiar relationships |
| Opportunities: Favourable or good conditions to do something. It can be states or situations that provides good position for success | Education | Any reference to the education system or the fact of study |
|  | Meritocracy beliefs | Endorse the idea that effort and hard work are enough to get ahead in life |
|  | Inequality of opportunities | Direct mentions of having unequal access to different opportunities that allow/obstacle the achievement of goals |
|  | Opportunities in life (general) | General mention to opportunities without specifying what kind of opportunities. |
| Poverty: State of having little or no money, goods, or means of support, or simply, not having enough of anything that is considered as necessary | Begging | References to people or to the fact of ask for money on the streets. |
|  | Homeless people | People who lives on the streets because having nowhere to live. |
|  | Poverty (general) | General mention to poverty without specifying any particular aspect |
| Affluence: Having abundance of money and material goods | Affluence or opulence (general) | State of having abundance of money or material goods, which might well be used to accomplish excessive desires |
|  | use of expensive goods and services | State or condition of appearing very wealthy by the use of goods and services that highlight the social status |

*(Continued)*

**Table S1.** *(Continued)*

| Category | Subcategories | Indicators |
| --- | --- | --- |
| Direction or senses: Subsidiary category to qualify the topics or categories identified. Thus, direction or senses try to complement the intention attributed to each category (when possible) | Economic activity | General mention to the economy of a region, city or any other place |
|  | Corruption | lack of transparency, mismanagement of resources, influence pedding, act out of the law, prioritise private interests over the public ones |
|  | Unequal access | Relevant differences in the quantity and quality of access to goods, services or any other things. Focus on the contrast of one group to another in relation to the things or services that can be accessed or used. |
|  | Inequality of physical conditions according to zones | Differences in the living conditions according to the socioeonomic stratification |
|  | Inequaility in living expenses | Living expenses variations from one side to another according to the sector of the city |
|  | Gender inequality | Any reference to unequal power relationships between men and women, as well as any other difference just because of gender reasons. |
|  | Income inequalities | Unequal monetary payment received for goods or services offered as part of an economic productive activity. |
|  | Misinformation | False information deliberately given for any media source; generally refers to misleading information |
|  | Lack or difficulties to access | Not having the possibility to get access to goods or services; focus on the idea of lack, absence, or not enough of something. Different from the Unequal access where the idea is centred on the contrast or comparison |
|  | Mobility difficulty | Struggling to ascend in the social ladder, difficulties to upward social mobility |
|  | Lack of economic resources | Not having enough money, cash, or economic resources in general |
|  | Privatization | References to the fact of privatize organisations, or to mention the role of private entities |
|  | Space segregation | Imposition of a separation of one group from the society; the fact to set apart groups in certain spaces |
|  | Discrimination | Prejudge people by making negative attribution on the basis of the group or social category to which people belongs. |

*(Continued)*

**Table S1.** *(Continued)*

| Category | Subcategories | Indicators |
| --- | --- | --- |
| Basic services: Main services that people should have in order to have a dignified life | Access to basic services (general) | General mention to minimum conditions to have a dignified life without specifying any particular service or good |
|  | Food | Aspects related to food security, decent access to a normal feeding |
|  | Health | Any mention to physical or mental health, also the health services, assistance to people regarding their health situation |
|  | Public transport | Mobility in the city means of transportation, not only public transport but any means to navigate through the physical space of the cities. |
|  | Housing | Housing, types of housing, physical space to live |
| Work: Related to the any economic  Productive activity through which people make a living. It includes both employment as other forms of work (cooperative, informal, independent, etc.) | Economic migration  Child labour | Go abroad to look for better labour opportunities  Economic activities that involved children or teenagers |
|  | Informal work | Economic activities out of the formality of employment, such as street vendor or any other expression of informal trade. |
|  | Career | Expectancies to advance in the professional career, or improve the occupational situation. |
|  | Unemployment | References to people or to the situation of not having employment. |
|  | Access to work | Related to the fact to get a job, or get access to any way of work (not only employment) |
|  | Work (general) | General mention to work without specifying anything in particular |
|  | Precarious work | Poor work conditions due to extended schedules, labour exploitation, low wages, or low occupational profile. |
| Free nodes: Free topics that raised during the analysis | Status anxiety | Feelings of anxiety regarding the expectancies of getting ahead in life |
|  | Evictions | To expel or force out a persona from a house, building, or similar. |
|  | Disability | Mentions to the lack of adequate ability to do something, anything that disables a persona at a disadvantage |
|  | Frequency perceiving inequality | Affirmation about the high prevalence of inequality |
|  | Motivation to get ahead in life | Desire, expectations, of achieving success in life |
|  | Life projects | The idea of having projects or dreams to achieve |
