# Supplementary material for: Perceptions of Economic Inequality in Colombian Daily Life: More Than Unequal Distribution of Economic Resources
Source: Front Psychol. 2018 Sep 6;9:1660. doi: 10.3389/fpsyg.2018.01660 (PMC6135891; doi:10.3389/fpsyg.2018.01660)
Supplement: Supplementary file 2 [file Table_2.DOCX]

Supplementary Material

**Perceptions of economic inequality in Colombian daily life: More than unequal distribution of economic resources**

**Efraín García-Sánchez, Guillermo B. Willis, Rosa Rodríguez-Bailón, Juan García-Castro, Jorge Palacio-Sañudo, Jean Polo, Erico Rentería-Pérez**

*** Correspondence:** Corresponding Author: [egarcias@correo.ugr.es](mailto:egarcias@correo.ugr.es)

| **Table S2.**  Frequencies and percentages of responses coded in each category | | |
| --- | --- | --- |
| **Category** | **Frequency**  **(Count)*** | **Percentage**  **(%)**** |
| Opportunities: Education | 304 | 18,72 |
| Actors: Social classes (includes elites and poor) | 274 | 16,87 |
| Living conditions: Income | 267 | 16,44 |
| Basic services: Health | 257 | 15,83 |
| Senses: Unequal access | 216 | 13,30 |
| Senses: Lack or difficulties to access | 192 | 11,82 |
| Actors: Workers | 179 | 11,02 |
| Living conditions: Public space | 163 | 10,04 |
| Senses: Discrimination | 150 | 9,24 |
| Actors: Elites | 139 | 8,56 |
| Senses: Income inequalities | 121 | 7,45 |
| Work: Precarious work | 119 | 7,33 |
| Actors: Poor | 96 | 5,91 |
| Interpersonal: Social comparisons | 95 | 5,85 |
| Interpersonal: Treatment of people | 94 | 5,79 |
| Living conditions: Socioeconomic stratification | 89 | 5,48 |
| Work: Work (general) | 85 | 5,23 |
| Senses: Corruption | 83 | 5,11 |
| Actors: University | 82 | 5,05 |
| Opportunities: Inequality of opportunities | 80 | 4,93 |
| Consuming: Consume products or services | 72 | 4,43 |
| Actors: Enterprises | 69 | 4,25 |
| Basic services: Mobility and transport | 68 | 4,19 |
| Senses: Lack of economic resources | 65 | 4,00 |
| Work: Informal work | 63 | 3,88 |
| Basic services: Food | 53 | 3,26 |
| Affluence: Affluence or opulence (general) | 52 | 3,20 |
| Work: Access to work | 48 | 2,96 |
| Senses: Space segregation | 44 | 2,71 |
| Actors: Children | 42 | 2,59 |
| Actors: Public entities | 41 | 2,52 |
| Actors: Public servants | 40 | 2,46 |
| Poverty: Begging | 39 | 2,40 |
| Basic services: Housing | 38 | 2,34 |
| Actors: Women | 37 | 2,28 |
| Senses: Privatization | 37 | 2,28 |
| Actors: Private entities | 36 | 2,22 |
| Actors: Directives, Chiefs, Bosses, supervisors | 31 | 1,91 |
| *(Continued)* | | |

| **Table S2.** *(continued)* | | |
| --- | --- | --- |
| **Category** | **Frequency**  **(Count)** | **Percentage**  **(%)** |
| Poverty: Homeless people | 31 | 1,91 |
| Consuming: Leisure | 30 | 1,85 |
| Senses: Inequality of physical conditions according to zones | 30 | 1,85 |
| Actors: Government | 29 | 1,79 |
| Living conditions: Living conditions (general) | 27 | 1,66 |
| Institutional: Public investment | 27 | 1,66 |
| Living conditions: Social subsidies | 22 | 1,35 |
| Institutional: Political system | 22 | 1,35 |
| Basic services: Access to basic services (general) | 22 | 1,35 |
| Poverty: Poverty (general) | 21 | 1,29 |
| Senses: Gender inequality | 21 | 1,29 |
| Living conditions: Criminality or insecurity | 20 | 1,23 |
| Work: Child labour | 20 | 1,23 |
| Work: Unemployment | 19 | 1,17 |
| Actors: Youth people | 18 | 1,11 |
| Interpersonal: Family | 18 | 1,11 |
| Senses: Mobility difficulty | 16 | 0,99 |
| Consuming: Queues to have access to services | 15 | 0,92 |
| Institutional: Taxes | 15 | 0,92 |
| Free nodes: Frequency perceiving inequality | 14 | 0,86 |
| Institutional: Justice | 13 | 0,80 |
| Actors: Peasants | 11 | 0,68 |
| Interpersonal: Social conflictiveness | 11 | 0,68 |
| Interpersonal: Ethnic or cultural issues | 11 | 0,68 |
| Opportunities: Meritocracy beliefs | 10 | 0,62 |
| Actors: Police | 9 | 0,55 |
| Actors: Banks | 8 | 0,49 |
| Living conditions: Economic resources concentration | 8 | 0,49 |
| Senses: Inequaility in living expenses | 8 | 0,49 |
| Actors: Private university | 7 | 0,43 |
| Living conditions: Forced displacement | 7 | 0,43 |
| Senses: Economic activity | 7 | 0,43 |
| Actors: Older people | 6 | 0,37 |
| Actors: Students | 6 | 0,37 |
| Living conditions: Predatory loans (banking or not) | 6 | 0,37 |
| Work: Career | 6 | 0,37 |
| Free nodes: Life projects | 5 | 0,31 |
| Living conditions: Pensions | 4 | 0,25 |
| Actors: Society (general) | 3 | 0,18 |
| Institutional: Media | 3 | 0,18 |
| Consuming: Saving | 2 | 0,12 |
| Free nodes: Disability | 2 | 0,12 |
| *(Continued)* | | |

| ***Table* S2*.*** *(continued)* | | |
| --- | --- | --- |
| **Category** | **Frequency**  **(Count)** | **Percentage**  **(%)** |
| Work: Economic migration | 2 | 0,12 |
| Living conditions: Rural sector | 1 | 0,06 |
| Free nodes: Status anxiety | 1 | 0,06 |
| Free nodes: Motivation to get ahead in life | 1 | 0,06 |
| Opportunities: Opportunities in life (general) | 1 | 0,06 |
| Affluence: use of expensive goods and services | 1 | 0,06 |
| * These counts are based on the number of responses that contain the indicated category | | |
| ** Percentage is computed based on the total amount of responses of our data corpus (N = 1624) | | |
