# Supplementary material for: Perceptions of Economic Inequality in Colombian Daily Life: More Than Unequal Distribution of Economic Resources
Source: Front Psychol. 2018 Sep 6;9:1660. doi: 10.3389/fpsyg.2018.01660 (PMC6135891; doi:10.3389/fpsyg.2018.01660)
Supplement: Supplementary file 3 [file Table_3.DOCX]

Supplementary Material

**Perceptions of economic inequality in Colombian daily life: More than unequal distribution of economic resources**

**Efraín García-Sánchez, Guillermo B. Willis, Rosa Rodríguez-Bailón, Juan García-Castro, Jorge Palacio-Sañudo, Jean Polo, Erico Rentería-Pérez**

*** Correspondence:** Corresponding Author: [egarcias@correo.ugr.es](mailto:egarcias@correo.ugr.es)

| **Table S3.** | | | | |
| --- | --- | --- | --- | --- |
| Network centrality measurements | | | |  |
| **Category** | **Subcategory** | **Degree Centrality** | **Betweenness centrality** | **Modularity class** |
| Actors | social classes | 61 | 169,30 | 1 |
| Actors | poor | 49 | 72,41 | 1 |
| Senses | discrimination | 45 | 83,93 | 1 |
| Actors | elites | 44 | 58,07 | 1 |
| Interpersonal | social comparison | 44 | 41,21 | 1 |
| Senses | corruption | 37 | 27,10 | 1 |
| Interpersonal | treatment of people | 34 | 25,87 | 1 |
| Affluence | Wealth or opulence | 31 | 14,18 | 1 |
| Basic services | access to basic services | 31 | 12,62 | 1 |
| Actors | government | 27 | 9,70 | 1 |
| Institutional | public investment | 24 | 6,22 | 1 |
| Institutional | political system | 20 | 8,39 | 1 |
| Work | access to work | 20 | 12,61 | 1 |
| Consumo | leisure | 19 | 1,60 | 1 |
| Institutional | justice | 18 | 4,02 | 1 |
| Interpersonal | ethnicity or culture | 18 | 2,95 | 1 |
| Interpersonal | social conflicts | 16 | 5,68 | 1 |
| Living cond. | wealth concentration | 15 | 2,64 | 1 |
| Institutional | taxes | 15 | 1,82 | 1 |
| Consumo | queues to access services | 14 | 3,27 | 1 |
| Senses | economic activity | 14 | 2,84 | 1 |
| Opportunities | meritocracy | 13 | 1,55 | 1 |
| Actors | banks | 8 | 0,37 | 1 |
| Affluence | use of expensive devices | 5 | 0 | 1 |
| Free nodes | disability | 3 | 0 | 1 |
|  |  |  |  | *(Continued)* |

| **Table S3.** (Continued) | | | | |
| --- | --- | --- | --- | --- |
| Network centrality measurements | | | |  |
| **Category** | **Subcategory** | **Degree Centrality** | **Betweenness centrality** | **Modularity class** |
| Living cond. | public space | 44 | 65,92 | 2 |
| Living cond. | socioeconomic stratification | 43 | 63,16 | 2 |
| Actors | childhood | 35 | 23,84 | 2 |
| Work | informal work | 35 | 37,86 | 2 |
| Senses | spatial segregation | 32 | 16,16 | 2 |
| Senses | inequality between strata | 30 | 9,84 | 2 |
| Consumption | consuming products or services | 28 | 22,31 | 2 |
| Poverty | poverty | 27 | 16,24 | 2 |
| Poverty | begging | 26 | 8,76 | 2 |
| Free nodes | inequality perception | 23 | 5,76 | 2 |
| Living cond. | insecurity or criminality | 22 | 6,94 | 2 |
| Interpersonal | family | 22 | 3,55 | 2 |
| Poverty | Homeless | 21 | 6,82 | 2 |
| Work | child labour | 17 | 1,43 | 2 |
| Senses | inequality living costs | 14 | 0,89 | 2 |
| Work | unemployment | 13 | 4,02 | 2 |
| Living cond. | forced displacement | 11 | 1,40 | 2 |
| Free nodes | get ahead in life | 11 | 0 | 2 |
| Institutional | media | 10 | 0,65 | 2 |
| Actors | elderly | 8 | 0,36 | 2 |
| Free nodes | status anxiety | 3 | 0 | 2 |
| Consumo | saving | 2 | 0 | 2 |
|  |  |  |  | (Continued) |

| **Table S3.** (Continued) | | | | |
| --- | --- | --- | --- | --- |
| Network centrality measurements | | | |  |
| **Category** | **Subcategory** | **Degree Centrality** | **Betweenness centrality** | **Modularity class** |
| Senses | lack/poor access | 59 | 217,53 | 3 |
| Senses | unequal access | 56 | 109,17 | 3 |
| Opportunities | education | 45 | 64,18 | 3 |
| Opportunities | Unequal opportunities | 43 | 44,74 | 3 |
| Basic services | health | 37 | 27,75 | 3 |
| Basic services | food | 36 | 33,73 | 3 |
| Living cond. | living conditions | 34 | 24,30 | 3 |
| Actors | university | 33 | 17,74 | 3 |
| Basic services | housing | 28 | 8,73 | 3 |
| Actors | public entities | 27 | 12,75 | 3 |
| Basic services | public transport | 27 | 7,14 | 3 |
| Senses | privatization | 25 | 9,06 | 3 |
| Actors | youth | 24 | 8,45 | 3 |
| Actors | private entities | 22 | 5,54 | 3 |
| Living cond. | social subsidies | 18 | 3,11 | 3 |
| Senses | mobility difficulties | 15 | 1,53 | 3 |
| Actors | students | 13 | 0,77 | 3 |
| Living cond. | predatory loans | 12 | 1,08 | 3 |
| Actors | private university | 11 | 0,13 | 3 |
| Free nodes | life projects | 9 | 0,25 | 3 |
| Living cond. | pensions | 6 | 0,06 | 3 |
|  |  |  |  | (Continued) |

| **Table S3.** (Continued) | | | | |
| --- | --- | --- | --- | --- |
| Network centrality measurements | | | |  |
| **Category** | **Subcategory** | **Degree Centrality** | **Betweenness centrality** | **Modularity class** |
| Senses | income inequalities | 13 | 6,52 | 4 |
| Living cond. | economic resources | 13 | 6,52 | 4 |
| Work | precarious work | 12 | 16,92 | 4 |
| Actors | workers | 12 | 4,52 | 4 |
| Actors | enterprises | 12 | 4,22 | 4 |
| Work | work | 11 | 2,55 | 4 |
| Senses | lack economic resources | 11 | 15,29 | 4 |
| Actors | women | 9 | 0,83 | 4 |
| Actors | managers | 9 | 0,83 | 4 |
| Actors | public servants | 8 | 0,80 | 4 |
| Work | career or mobility | 6 | 0 | 4 |
| Senses | gender inequality | 6 | 0 | 4 |
| Actors | police | 5 | 0 | 4 |
| Actors | peasants | 5 | 0 | 4 |
| Work | work migration | 1 | 0 | 4 |
| Opportunities | opportunities in life | 1 | 0 | 4 |
| Note: Modularity class correspond to:  1 = Social classes and intergroup relations  2 = Public space and social exclusion  3 = Inequality of opportunities  4= Work and income inequalities | | | | |
